# s-CRIq: the online short version of the Cognitive Reserve Index Questionnaire

**DOI:** 10.1007/s40520-023-02561-1

**Published:** 2023-09-21

**Authors:** Sara Mondini, Veronica Pucci, Massimiliano Pastore, Ombretta Gaggi, Pier Paolo Tricomi, Massimo Nucci

**Affiliations:** 1 Department of Philosophy, Sociology, Education and Applied Psychology, FISPPA, University of Padua, Padua, Italy; 2 Human Inspired Technology Research Centre, University of Padua, Padua, Italy; 3 Department of Developmental Psychology, DPSS, University of Padua, Padua, Italy; 4 Department of Mathematics, University of Padua, Padua, Italy; 5 Department of General Psychology, DPG, University of Padua, Padua, Italy

**Keywords:** Cognitive Reserve, Lifestyle, Education, Working activity, Leisure-time

## Abstract

**Background:**

The wide use of the term Cognitive Reserve (CR) is in need of a clear and shared definition of its concept and of the development of new tools, quick and easy to use and updated for the people of today. This study describes the online short CRIq (s-CRIq), the new shorter version of the CRIq, following an item analysis revision, and compares the data distribution of different samples.

**Methods:**

The s-CRIq was administered online to 435 people while another 440 filled out the s-CRIq in self-administration. A further 588 participants had been administered the original paper-and-pencil long CRIq and 344 the online long CRIq.

**Results:**

The major difference in the databases of s-CRIq versus the long versions is an increased score in education and in leisure activity. However, the density distributions of the total score of CRI in the 4 databases share 64% of their areas, and at least two of them share 84%.

**Conclusion:**

The s-CRIq proved to be a simple and easy-to-administer tool. Similarly, to the original version, the s-CRIq is freely available on the web, and it is our hope that it will be of fruitful use for researchers and clinicians alike.

**Supplementary Information:**

The online version contains supplementary material available at 10.1007/s40520-023-02561-1.

## Introduction

According to SCOPUS the term “Cognitive Reserve” (CR) was included either in the title or in the abstract or was among the keywords of 321 articles in 2022 (search run on February 9, 2023). On average, this is almost one per day. This extraordinary success stems from a few ideas which had been left unnoticed for quite some time. The CR construct was prefigured in the late Sixties [1] of the past century and, later on, it developed in the area of Alzheimer's disease (AD) during the Nineties [2–4]. The development of the CR concept faced many challenges, especially the fact that the surprising clinical heterogeneity of people with AD is connected also to their formal education and the continuous and coordinated use of cognitive abilities throughout their life.

In this century, CR has found its first effective definitions and applications and, since the Twenties, many studies on CR or using CR have been recorded. The CR construct has been exported - with increasing success - to areas that involve other pathological conditions, such as Parkinson’s disease [5], multiple sclerosis [6], traumatic brain injury [7, 8], stroke [9], psychiatric disorders [10], and, not least, healthy cognitive ageing [11], and adolescence [12]. The increasing number of authors who have used and developed the concept of CR has led to a proliferation of terms. Recently, a laudable initiative (https://reserveandresilience.com) has promoted a series of meetings to collaborate on a consensus document collecting shared definitions.

According to Stern, who contributed most to the development of the CR concept, the “reserve is defined as the adaptability (i.e., efficiency, capacity, flexibility) of cognitive processes that helps explain the differential susceptibility of cognitive abilities or day-to-day function to brain ageing pathology or insult” [13] (p. 1306: consensus document). Updates on the state of the art of CR come from numerous recent reviews and meta-analyses, for example, related to healthy older adults [14], to MCI [15], to dementia [16], to stroke [10], to schizophrenia [10], or to neuroimaging [17].

Besides the diffusion of CR studies, research on its quantitative estimation has developed, beginning with the study of the exact nature of the concept, which in turn is found in the epistemological status of the underlined models. CR is almost unanimously considered a latent variable, that is, a variable that is neither directly observable nor measurable. However, according to Borsboom [18] and to Jones [19], the ontological status of a latent variable can be distinguished into two categories: the first is the *realist,* in which the latent variable exists independently from its measurement; the second is the *anti-realist,* in which the latent variable does not exist without its measurement. Borsboom [20] argues convincingly that the only epistemologically scientifically sustainable position is the *realist* one. Thus, the relationship between the construct and its proxies becomes crucial and again, two models can be distinguished: reflective and formative [21]. In the so-called reflective model (much used in psychology, e.g., the measurement of intelligence) the indicators are reflections - direct effects - of the latent variable, whereas in the so-called formative model (more frequent in sociology or economics, e.g., measurement of socio-economic status) the latent variable is the effect of the proxy and not its cause. For example, the cost of your car is a “reflection“ of your socio-economic status, whereas the salary is “formative” of your socio-economic status. However, nowadays, there are no elements in favour of either model for the estimation of CR [22].

Continuing with the construct development, it is necessary to take into account the relationships that CR has with neighbouring constructs. In fact, since the beginning of the Twenties, the number of definitions and concepts proposed for CR has been incredibly high, ranging from simple synonyms to partially overlapping or flanking terms [23]. All these attempts aim to complete, circumscribe or assimilate CR to other already known concepts.

Nowadays, it can be stated in line with the consensus document [24] that “cognitive reserve”, “brain reserve” and “brain maintenance” are the concepts that exhaustively describe our field of investigation and those around which there is the greatest consensus.

Finally, a recent review [25] focusing on the main characteristics and psychometric properties of CR instruments has cited the most commonly used questionnaires in its esteem. Among these is the Cognitive Reserve Index Questionnaire (CRIq [26]; freely available on www.cognitivereserveindex.org), a tool to measure CR published by the same research group who is writing this paper. CRIq has been largely used in both clinical settings and in research: up to now CRIq has been translated into and in some cases adapted into 19 different languages at least (see details on CRIq diffusion in Supplementary Materials 1).

In the toolkit of any clinical psychologists and researchers, online instruments are always more present. They allow to obtain more efficient measurements, more secure storage of data and, in general, they allow to work in a more sustainable way. More than ten years after the publication of CRIq [26], it is desirable to have a fully (the administration and the data storage) digitalised version of the questionnaire and an updated normative sample. Thus, we have designed a new, online, self-administered, short version of the questionnaire, named the short Cognitive Reserve Index Questionnaire, from here on s-CRIq.

The aim of this paper is to present this revised version of CRIq and compare its performance with that of the other previous paper-and-pencil and online versions of the CRIq.

### s-CRIq: the new online short version of the Cognitive Reserve Index Questionnaire

The CRIq is a questionnaire [26] that collects and quantifies the main cognitively stimulating activities a person has carried out during their adult life. CRIq is a composite tool that evaluates education, working activities and leisure time activities measured on three sub-indexes: CRI-Education, CRI-WorkingActivity, and CRI-LeisureTime. The global score, Cognitive Reserve Index is the average of these three. In the new online short version, the CRI-Education and the CRI-WorkingActivity are equivalent to the original CRIq. However, in the new s-CRIq, the online administration allows a more agile collection of this information. CRI-Education is recorded by directly selecting the level achieved (without having to calculate years of education). Similarly, the occupation (or occupations, if there were more than one) in the s-CRIq are directly written and selected from an extensive database of about 6000 jobs derived from the International Standard Classification of Occupation (ISCO-08 [27]), which automatically classifies into five classes according to the cognitive load and responsibility involved (see Supplementary Materials 2).

The main difference between the CRIq and the s-CRIq is the number of items in the part of the questionnaire related to leisure time activities (CRI-LeisureTime). Of the original 17 activities recorded by the CRIq, the s-CRIq records only 5 (see “s-CRIq item selection”). To these five items, a new one has been added, which includes some activities from three of the original items (in Table 1 all the six items are reported, for the 17 original items see the links below).

**Table 1:** Table shows the 5 items selected from the original CRIq for the s-CRIq plus the added new one (item 2)

Items of CRI-LeisureTime	
1	Have you ever read newspapers or magazines (no social network) at least three times a week?
2	Have you ever spent your free time in leisure activities (for example sports, artistic activities, board games, crafts, gardening, puzzles, embroidery, photography, etc.) at least three times a week?
3	Have you ever attended conferences, concerts or exhibitions at least three times a year?
4	Have you ever taken trips or holidays lasting more than one day at least three times per year?
5	Have you ever read books, at least three per year?
6	Do you have children?

## Methods and materials

Data, data analyses and the link for the online administration of s-CRIq (Italian and English versions) are freely available at the OSF link https://osf.io/efzhs/. The link mentioned above (www.cognitivereserveindex.org) is also available at the original website. All the analyses were conducted with R [28].

### s-CRIq Item selection in CRI-LeisureTime

The CRI-LeisureTime sub-section comprises only six items, five of them selected from the original seventeen items of the CRIq, plus a new one. The selection procedure is grounded on two different approaches: Item Response Theory and Confirmatory Factor Analysis. Both these methods yield a score which estimates the capability to detect the construct of interest. The five items selected for the s-CRIq (see Table 2 above) were consistently the highest scores in both methods (for more details see Supplementary Material 3).

**Table 2:** Table shows the main characteristics of the four databases (ALP, ALO, ASO and SSO)

Administration	Administered		Administered	Self-administered	
Length	Long CRIq		Short CRIq		
Version	Paper	Online	Online		
Time interval	2008–2010	2018–2019	2021–2022	2021–2022	2008–2022
Database name	ALP	ALO	ASO	SSO	Tot
Sample size (*N*)	588	344	435	440	1807
Male/Female (%)	55/45	60/40	59/41	65/35	59/41
Age M (SD)	50.2 (19.7)	50.2 (21.3)	51.9 (19.7)	44.6 (18.3)	49.3 (19.8)
18–37 (*N*)	174	122	125	180	601
38–57 (*N*)	217	85	127	158	587
58–77 (*N*)	124	83	132	80	419
≥ 78 (*N*)	73	54	51	22	200

*ALP* Administered, Long, Paper-and-Pencil CRIq; *ALO* Administered, Long, Online CRIq; *ASO* Administered, Short, Online CRIq; *SSO* Self-administered, Short, Online CRIq

### s-CRIq scores calculation

The algorithm to compute the s-CRIq scores is the same as in the original version^1^ The raw score of each section of the s-CRIq is collected and counted as follows:

- Education: years of education are automatically recorded according to the level of schooling achieved. In addition, any other structured course lasting at least six months is included.
- Working activity: years of occupation are recorded in one of five classes according to the cognitive load and responsibility involved (in line with the ISCO-08 classification). Only the three most relevant working activities are considered for the scoring.
- Leisure time: number of years that six leisure activities are carried out continuously for a minimum of one year. Depending on the type of activity, also its frequency -weekly, monthly or yearly- is taken into account.

The three sub-scores of the s-CRIq (CRI-Education, CRI-WorkingActivity and CRI-LeisureTime) are the residuals of the three corresponding simple linear regressions, where the raw score is the dependent variable and the individual's age is the independent variable (i.e., the predictor). For sake of readability, the residuals were then standardised and transposed into a scale with M=100 and SD=15. Finally, the total score of s-CRIq, named Cognitive Reserve Index (hereafter CRI), was the average of the three sub-scores, again standardised and transposed to a scale with M=100 and SD=15. The result is that the higher the CRI score, the higher the estimated CR. CRI scores are conventionally classified into 5 ordered levels: Low (<70), Medium-low (70–84), Medium (85–114), Medium-high (115–130) and High (more than 130). All details about the rationale of s-CRIq calculation are reported in Supplementary Material 4.

### Data collection and databases

Data collection was carried out at different intervals of time. From 2008 to 2010 data were gathered by some of the authors of this paper exclusively via the original paper-and-pencil questionnaire as a semi-structured interview. This first database is the one on which the questionnaire was initially built [26] and it was the normative database of the CRIq. We named this original database: Administered Long Paper (from here on ALP). From 2018 to 2019 postgraduate and master students in psychology at the University of Padua administered the same CRIq as a semi-structured interview to a new sample of Italian participants, but this time data were collected through the online version of the questionnaire. We named this database: Administered Long Online (from here on ALO). Recently, from 2021 to 2022 postgraduate and master students in psychology at the University of Padua administered the s-CRIq to a new sample of participants by means of the short online version of the questionnaire. We named this database: Administered Short Online (from here on ASO). Finally, at the same time, these students invited some of their acquaintances (never tested before) to fill in the online s-CRIq in self-administered modality. We named this last database: Self-administered Short Online (from here on SSO). Table 2 below shows the main characteristics of all these four databases.

## Results

### Descriptive statistics and distribution overlapping

Databases differ according to the modality of administration of the questionnaire (Administered by an expert or Self-administered), length (number of items; Long or Short) and the version (Paper-and-pencil or Online). The whole sample from the four databases included 1807 individuals (statistical units). On each individual, total CRI and the three sub-indexes were calculated using the original parameters of the first data collection (i.e., ALP; new norms of CRI-LeisureTime were calculated only on the five items shared by all databases). The aim was to compare the different databases using descriptive indexes and their empirical shape distributions of data. Table 3 below shows the main descriptive indexes of the four databases considered and compared in the present paper. To quantify the similarity between the empirical shape distributions, we considered the area intersected by empirical density functions quantified through the Overlapping Index [29, 30].

**Table 3:** Table compares means and standard deviations of CRI and CRI sub-scores of the four databases

Administration	Administered		Administered	Self-administered
Length	Long CRIq		Short CRIq	
Version	Paper	Online	Online	
Time collection	2008–2010	2018–2019	2021–2022	
Database name	ALP	ALO	ASO	SSO
CRI-Education	100 (15)	102.4 (14.0)	103.1 (15.9)	107.7 (17.7)
CRI-WorkingActivity	100 (15)	100.6 (15.2)	97.8 (15.7)	101.8 (14.8)
CRI-LeisureTime	100 (15)	106.6 (20.2)	109.3 (20.8)	112.7 (21.2)
CRI	100 (15)	104.2 (17.3)	104.4 (17.2)	109.7 (17.9)

*ALP *Administered, Long, Paper-and-Pencil CRIq; *ALO* Administered, Long, Online CRIq; *ASO* Administered, Short, Online CRIq; *SSO* Self-administered, Short, Online CRIq

### CRI-Education

**Fig. 1:**
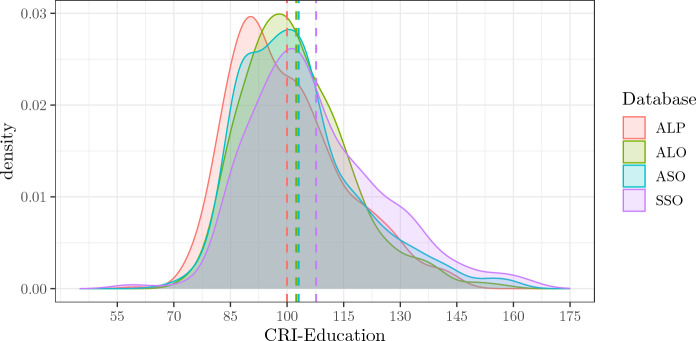
Figure shows the empirical density distributions of CRI-Education of the four databases. Dotted vertical lines signal the means of each distribution. *ALP* Administered, Long, Paper-and-Pencil CRIq, *ALO* Administered, Long, Online CRIq, *ASO* Administered, Short, Online CRIq, *SSO* Self-administered, Short, Online CRIq

The empirical density distributions of CRI-Education across the four databases show substantial uniformity, with a fairly pronounced positive skew. As expected, the more recent the databases, the more the mean and the mode of the distributions are right-shifted (see Fig. 1 and Table 3), indicating a steady increase in the level of schooling of the population. The SSO sample shows a higher mean and a more pronounced right tail than the other databases. Overall, all four empirical density distributions share 64% of their areas, and at least two of them share 84%.

### CRI-WorkingActivity

**Fig. 2:**
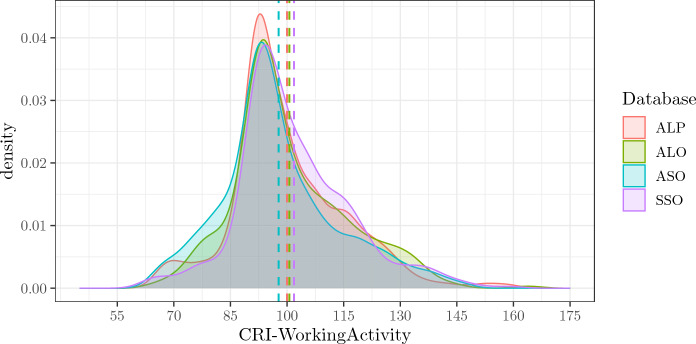
Figure shows the empirical density distributions of CRI-WorkingActivity of the four databases. Dotted vertical lines signal the means of each distribution. *ALP* Administered, Long, Paper-and-Pencil CRIq, *ALO* Administered, Long, Online CRIq, *ASO* Administered, Short, Online CRIq, *SSO* Self-administered, Short, Online CRIq

The empirical density distributions of CRI-WorkingActivity are the most similar between the four databases, as they share an almost equivalent mode and very similar mean and shape (see Figure 2 and Table 2). The more recent SSO and ASO databases show slightly different characteristics, with SSO the highest and ASO the lowest mean scores in the four databases. Overall, all four empirical density distributions share 70% of their areas, and at least two of them share 87%.

### CRI-LeisureTime

**Fig. 3:**
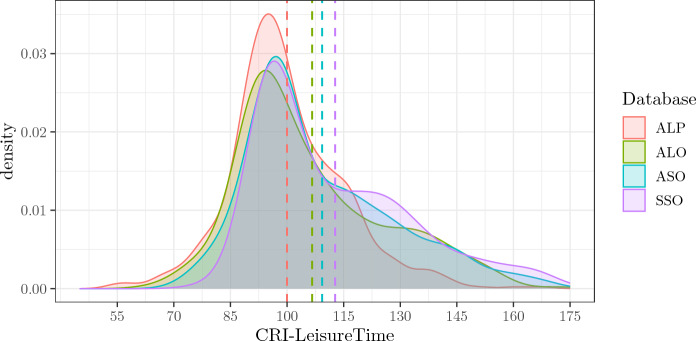
Figure shows the empirical density distributions of CRI-LeisureTime of the four databases. Dotted vertical lines signal the means of each distribution. *ALP* Administered, Long, Paper-and-Pencil CRIq, *ALO* Administered, Long, Online CRIq, *ASO* Administered, Short, Online CRIq, *SSO* Self-administered, Short, Online CRIq

The empirical density distributions related to CRI-LeisureTime (this index was calculated on the five items of the s-CRIq in all four databases) differ the most. The means of the four distributions differ the most from one another (see Fig. 3 and Table 2). As in the case of CRI-Education, the most recent databases are shifted to the right, and the distance between means is very noticeable especially compared to the original database (ALP). Remarkably, however, CRI-LeisureTime gives rise to distributions with the most substantial positive skew. In particular, the differences are clear and consistent for the three most recent databases (ALO, ASO and SSO) compared to the original one (ALP). Overall, all four empirical density distributions share 60% of their areas, and at least two of them share 87%.

### Total CRI

**Fig. 4:**
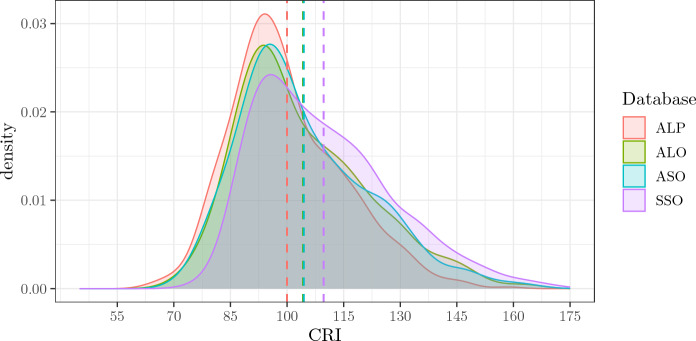
Figure shows the empirical density distributions of CRI Total score of the four databases. Dotted vertical lines signal the means of each distribution. *ALP* Administered, Long, Paper-and-Pencil CRIq, *ALO* Administered, Long, Online CRIq, *ASO* Administered, Short, Online CRIq, *SSO* Self-administered, Short, Online CRIq

As might be expected, the empirical density distributions of the CRI in the four databases summarise those observed in the three sub-indexes. The mean shows a trend to increase in the more recent databases from ALP to SSO, although ALO and ASO are almost equivalent. The shape of the distributions appears broadly overlapped, with marked positive skewness and nearly equivalent mode in the four distributions. Overall, all four empirical density distributions share 65% of their areas, and at least two of them share 86% (Fig. 4).

## Discussion

This paper introduces the short Cognitive Reserve Index Questionnaire (s-CRIq), an instrument devised to collect and estimate the individual, cognitive-stimulating life-experience activities of a person. The s-CRIq has several advantages: it is shorter than the original (CRI-LeisureTime is reduced from 17 to 6 items), more user-friendly, intuitive and fast to administer, it can be filled out completely online and can be self-administered. Thus, it could be adopted extensively and routinely in both research and clinical settings. In this regard, a promising use of s-CRIq is in interpreting test scores. Montemurro et al. [31] recently showed that adding CR to age and education when adjusting test scores improves the accuracy of cut-offs in the Montreal Cognitive Assessment [32] in healthy older adults. Also, two new cognitive tests, the Global Examination of Mental State (GEMS [33]) and the “Esame Neuropsicologico Breve 3” (tr. Brief Neuropsychological Examination 3 [34]) allow considering s-CRIq when comparing performance within the normative sample. Using the s-CRIq in self-administration modality is an obvious time saver, although this should be used cautiously in a clinical setting. Indeed, a patient may find it hard to understand or to answer the questions and thus their responses may be inaccurate or completely wrong. In these cases, the semi-structured interview administered by an expert is highly recommended.

Next to the development of the s-CRIq, the databases collected over time using the different forms of the questionnaire (Administered vs. Self-administered, Long vs. Short, Paper vs. Online) allow us to compare how some variables of interest change over time in the Italian population. From the first database (ALP [26]) to the most recent ones (ALO, ASO, SSO), the main change is related to education and leisure time. In particular, education progressively increases in the population, and this reflects a general trend in Italian society, where in 2007 mandatory schooling was extended from 14 to 16 years of age. Similarly, leisure time shows a progressive increase in the mean of the scores across databases, whereas variability is more pronounced in the recent ones. This change can be explained by the increased well-being experienced by people in Italy, the greater availability of activities and the fact that, nowadays, they are more and more embedded in daily-life routines. Unexpectedly, working activity scores do not show a comparable increase and means and shapes are the most similar between databases. CRI total score also shows a marked overlap between distributions, with a slight right-shifting of the average scores. It is noteworthy that the most recent database (SSO), in all the sub-indexes, shows a modest but appreciable different pattern in the distribution shape. In particular, its right tail (highest estimated CR) is systematically higher than the right tails of the other databases. At least two factors may explain this outcome: first, this most recent sample includes the youngest individuals and, second, it might be a not fully-randomised sample, meaning that, in all probability, recruitment of these individuals was biassed towards high-functioning people, able to fill out the online questionnaire without any trouble. These observed changes justify the updating of the normative sample considering only the more recent databases (i.e., ALO, ASO, SSO). The new parameters for CRI calculation are available on the website, and they will be regularly updated in accordance with the new data gathered through the website itself.

The clinical and research use of CR construct will likely become more widespread in different countries. So far, CRIq has been translated into a number of languages, as reported in Supplementary Materials 3. However, as disciplines like “Cultural psychology” and “Cultural neuropsychology” have underlined, there is substantial evidence that a person’s cultural background and place of origin influence cognition and performance in cognitive tests [35]. This is even more so as far as the CRIq is concerned, as it collects life experiences which can be very different across cultures and times. The new s-CRIq is based on data from the current Italian population and it is representative since it reflects today’s life experiences typical of this European country. Thus, before using s-CRIq in a new language it should be not simply translated but also adapted to that culture, and normative data collection would also be necessary (see for example Maiovis [36] for the Greek CRIq).

Like any new instrument, s-CRIq is not exempt from limitations. The small number of items compared to the long version of CRIq may reduce accuracy. In particular, this is more evident when the CR score falls on the tails of the distribution (i.e., people with very low or very high estimated CR). Moreover, it is reasonable to think that slight differences may emerge in the case of self-administration. When people evaluate themselves they seem to be more indulgent and tend to overestimate the extent and richness of their life experiences. The informatization of data collection will allow comparing results and provide different norms for each version.

In conclusion, the s-CRIq clearly allows the quick and effective collection of an individual’s detailed and comprehensive personal history. This can be useful in research and in clinical settings, both for better quality of assessment and for more personalised treatment promoting precision medicine.

### Supplementary Information

Below is the link to the electronic supplementary material.

Supplementary file1 (PDF 118 KB)

Supplementary file2 (PDF 35 KB)

## Data Availability

Data and data analyses are freely available at the OSF link https://osf.io/efzhs/.

## Footnotes

1 The only exception is a small adjustment in the computation of CRI-WorkingActivity when there are more than two jobs: in the old version, adding more than two jobs could lead to a small—paradoxical—reduction in the score. In the new version, this flaw in the algorithm has been corrected.
