# Supplementary material for: s-CRIq: the online short version of the Cognitive Reserve Index Questionnaire
Source: Aging Clin Exp Res. 2023 Sep 21;35(12):2903–10. doi: 10.1007/s40520-023-02561-1 (PMC10721653; doi:10.1007/s40520-023-02561-1)
Supplement: Supplementary file 1 — Supplementary file1 (PDF 118 KB) [file 40520_2023_2561_MOESM1_ESM.pdf]

SUPPLEMENTARY MATERIALS

s-CRIq: the online short version of the Cognitive Reserve Index questionnaire

Sara Mondini, Veronica Pucci, Massimiliano Pastore, Ombretta Gaggi, Pier Paolo Tricomi and Massimo Nucci

1. Diffusion of the CRIq

In the spirit of open science, the original CRIq has always been available as open access, and it can be used for free but cannot be substantially modified or used for financial gain. The large national and international acknowledgement of CRI-q helpfulness is also due to its being easily accessed through the website www.cognitivereserveindex.org. Over time, we have been contacted by several research groups who translated into their own language and, in some cases, adapted the original CRIq. Most of these versions are available on the website. Supplementary Table 1 lists the different translations and adaptations, the sample size of normative data, and, when available, the actual paper.

Supplementary Table 1. Table shows all the CRIq translated and/or adapted, listed in alphabetical order by language.

#	Language	Adaptation	Normative data (yes/no); sample size	Authors and year	Availability on website
1	Arabic (Egypt)	no	no	Tr. by Abedl Rahman - unpublished	no
2	Bulgarian	no	yes; N=114	Yaneva et al., 2019[1]	no
3	Catalan	no	no	Tr. requested by authors of CRIq	yes
4	Chinese	no	yes; N=371	Cao et al., 2022[2]	no
5	Czech	no	no	Tr. by Vil´ımovsk´y, 2016 - unpublished	yes
6	Dutch	no	no	Tr. by Kessels & Oosterman 2016 – unpublished	yes

7	English	no	no	Tr. by Jarema & Kehaya - unpublished	yes
8	French	no	no	Tr. by Jarema & Kehaya - unpublished	yes
9	German	no	no	Tr. requested by authors of CRIq	yes
10	Greek	yes	yes; N=591	Maiovis et al., 2016[3]	yes
11	Hungarian	no	no	Tr. by Bozzai & Karádi - unpublished	yes
12	Indonesian	no	yes; N=66	Kusumaningrum et al., 2021[4]	no
13	Korean	NA	yes; N=358	Choi et al., 2016[5]	no
14	Latvian	no	no	Tr. by Sneidere & Harlamova, 2017 - unpublished	yes
15	Malay	no	yes; N=80	Abdullah et al., 2021[6]	no
16	Persian	no	yes; N=385	Hatami et al., 2020[7]	no
17	Portuguese	no	no	Tr. requested by authors of CRIq	yes
18	Russian	no	no	Tr. by Кутиков & Никишкова, 2020 - unpublished	yes
19	Serbian	yes	yes; N=117	Volarov et al., 2020[8]	yes
20	South African (isiZulu)	yes	no	Narsi, Tomita & Ramlall, 2020	no
21	Spanish	no	no	Tr. requested by authors of CRIq	yes
22	Turkish	no	yes; N=499	Ozakbas et al., 2021[9]	yes
23	Turkish	yes	yes; N=175	Çebi & Kulce, 2021[10]	yes

2. International Standard Classification of Occupation (ISCO)

The classification used for the scoring of CRI-WorkingActivity is fully supported by the international community as an accepted standard for international labour statistics in Europe. The system classifies each working activity with numbers on a continuum, where the higher the number the lower the complexity of the occupation in terms of skills and cognitive resources involved (ranging from 1111 to 9629; e.g., 3153 is for “astronaut”, 7533 is for a “toy maker” and 9333 is for “porter”). The s-CRIq still classifies each job into 5 levels of cognitive load, as in the original CRIq, but the occupation is classified into one of them automatically.

3. s-CRIq Item selection procedure

The s-CRIq has been developed from the reduction of the items of CRI-LeisureTime from 17 to 6, i.e. 5 selected from the original CRIq and one added. The item selection was based on data collected in 2019 using the Administered Long Online CRIq (ALO database). The selection procedure is grounded on two different approaches: Confirmatory Factor Analysis and Item Response Theory. Both these methods yield a score which estimates how capable an item is to detect the construct of interest. The 5 items selected for the s-CRIq (see Table 1 in the manuscript) were those with the highest scores in both methods (see Supplementary Figure 1, and Supplementary Table 2).

A new item has been added to the five selected ones. It is related to activities carried out during free time, chosen by 92 individuals in an informal survey. The same activities had all been described separately in the original version of the questionnaire, while in the s-CRIq they are all included as examples in the new item.

Supplementary Table 2. Table shows the values of R^2 (deriving via Confirmatory Factor Analysis) and of β (deriving via Item Response Theory). The two approaches were used to select the best items of the CRI-LeisureTime section of CRIq: the 5 items with highest scores in both approaches were chosen for the s-CRIq (in bold).

		Confirmatory Factor Analysis approach	Item Response Theory approach
		R^2	β
**Item 1**	**Newspapers**	**0.400**	**1.401**
Item 2	Household	0.247	0.767

Item 3	Driving	0.254	0.946
Item 4	Hobby/sports	0.253	0.864
Item 5	New technologies	0.047	0.510
Item 6	Social activities	0.133	0.567
Item 7	Cinema/theatre	0.233	0.927
Item 8	Caring	0.111	0.424
Item 9	Gardening	0.192	0.773
Item 10	Volunteering	0.105	0.777
Item 11	Artistic activities	0.091	0.464
**Item 12**	**Concerts/conferences**	**0.292**	**1.251**
**Item 13**	**Travelling**	**0.370**	**1.461**
**Item 14**	**Books**	**0.488**	**2.109**
**Item 15**	**Children**	**0.066**	**0.639**
Item 16	Pet	0.071	0.291
Item 17	Bank account	0.390	1.248

Supplementary Figure 1. Figure shows the items selected for the s-CRIq via the two statistical approaches (Confirmatory Factor Analysis and Item Response Theory). On the x-axis is reported the β of IRT, and on the y-axis is reported the R^2 of the CFA. All items above the dotted line are the chosen ones.

Supplementary Figure 1,
here
----

4. The rationale for CRI calculation

The CRIq scoring is based on three linear models. Knowing the parameters of the models and the age of the person, it is possible to estimate the best-predicted value of the raw score for each of the sections of the CRIq (the values are obviously the same for people the same age). The residual (i.e., the difference between the predicted and the observed values) is the score of each of the three sub-indices of the CRIq. Choosing scores as residuals of the linear model has at least two advantages. The first is that the scores are not correlated with age, although they are closely linked to the number of years in which the activities have been carried out (as is well known, residuals are not correlated with predictors). For example, a CRI-Education of 110 gives the same information about either

a 30-year-old individual and 80-year-old individual. In other words, the CRIq scores quantify the frequency and regularity of the engagement of cognitive abilities over time, regardless of age. An effect of this is that the variability of CRIq scores in the population grows as age increases (at the age of 20, people have very similar CRI, whereas at 80 the CRI score can be very different). A second advantage of using the linear model is that a person is compared with individuals of the same generation, that is those who share the same "world", that is, the historical, socio-economic and cultural background where the person has lived. For example, a person with just eight years of education, reached at a time in which it was hard to go to school (because of war-time or other social issues), has a high CRI-Education score. On the other hand, a person with the same years of schooling, but achieved in a period in which everyone averaged at least 10 years, has a low CRI-Education score. This same reasoning can be applied to the other two sub-indices. Total CRI is the result of the average of the three sub-indexes without giving any more information.

References

[1] A. Yaneva, R. Massaldjieva, N. Mateva, and D. Bakova, "Assessment of Cognitive Reserve: a pilot study for Bulgarian Population," *Eur. J. Public Health*, vol. 29, no. Supplement_4, p. ckz186.578, Nov. 2019, doi: 10.1093/eurpub/ckz186.578.

[2] T. Cao, S. Zhang, M. Yu, X. Zhao, and Q. Wan, "The Chinese Translation Study of the Cognitive Reserve Index Questionnaire," *Front. Psychol.*, vol. 13, 2022, doi: 10.3389/fpsyg.2022.948740.

[3] P. Maiovis, P. Ioannidis, M. Nucci, A. Gotzamani-Psarrakou, and D. Karacostas, "Adaptation of the Cognitive Reserve Index Questionnaire (CRIq) for the Greek population.," *Neurol. Sci. Off. J. Ital. Neurol. Soc. Ital. Soc. Clin. Neurophysiol.*, vol. 37, no. 4, pp. 633–636, Apr. 2016, doi: 10.1007/s10072-015-2457-x.

[4] P. Kusumaningrum, D. Andoko, C. E. Damping, and M. W. S. Nasrun, "The Role of Cognitive Reserve as Measured by Cognitive Reserve Index Questionnaire Indonesia Version in Geriatric Delirium Cases," *eJournal Kedokt. Indones.*, vol. 9, no. 1 SE-Research Article, p. 44, May 2021, doi: 10.23886/ejki.9.29.44.

[5] C. H. Choi, S. Park, H.-J. Park, Y. Cho, B. K. Sohn, and J.-Y. Lee, "Study on Cognitive Reserve in Korea Using Korean Version of Cognitive Reserve Index Questionnaire," *J Korean Neuropsychiatr Assoc*, vol. 55, no. 3, pp. 256–263, Aug. 2016, [Online]. Available: https://doi.org/10.4306/jknpa.2016.55.3.256.

[6] A. H. Abdullah, S. Sharip, A. H. A. Rahman, and L. Bakar, "Cognitive reserve

in stroke patients," *PsyCh J.*, vol. 10, no. 3, pp. 444–452, 2021, doi: https://doi.org/10.1002/pchj.423.

[7] J. Hatami, A. Feizi khajeh, A. Rahiminezhad, H. Farahani, and T. Rezapour, "Validation of Persian Version of Cognitive Reserve Index Questionnaire (CRIq)," *J. Appl. Psychol. Res.*, vol. 11, no. 1, pp. 87–99, 2020, doi: 10.22059/japr.2020.288425.643348.

[8] M. Volarov *et al.*, "Serbian cognitive reserve index questionnaire: Adaptation and validation," 2020, [Online]. Available: http://www.icf.fasper.bg.ac.rs/zbornici/20200825_1-Thematic-Collection-2020.pdf.

[9] S. Ozakbas *et al.*, "Validity and reliability of 'Cognitive Reserve Index Questionnaire' for the Turkish Population.," *Mult. Scler. Relat. Disord.*, vol. 50, p. 102817, May 2021, doi: 10.1016/j.msard.2021.102817.

[10] M. Çebi and S. N. Kulce, "The Turkish translation study of the Cognitive Reserve Index Questionnaire (CRIq).," *Appl. Neuropsychol. Adult*, vol. 29, no. 6, pp. 1536–1542, 2022, doi: 10.1080/23279095.2021.1896519.
