# Supplementary figures and images for: s-CRIq: the online short version of the Cognitive Reserve Index Questionnaire
Source: Aging Clin Exp Res. 2023 Sep 21;35(12):2903–10. doi: 10.1007/s40520-023-02561-1 (PMC10721653; doi:10.1007/s40520-023-02561-1)

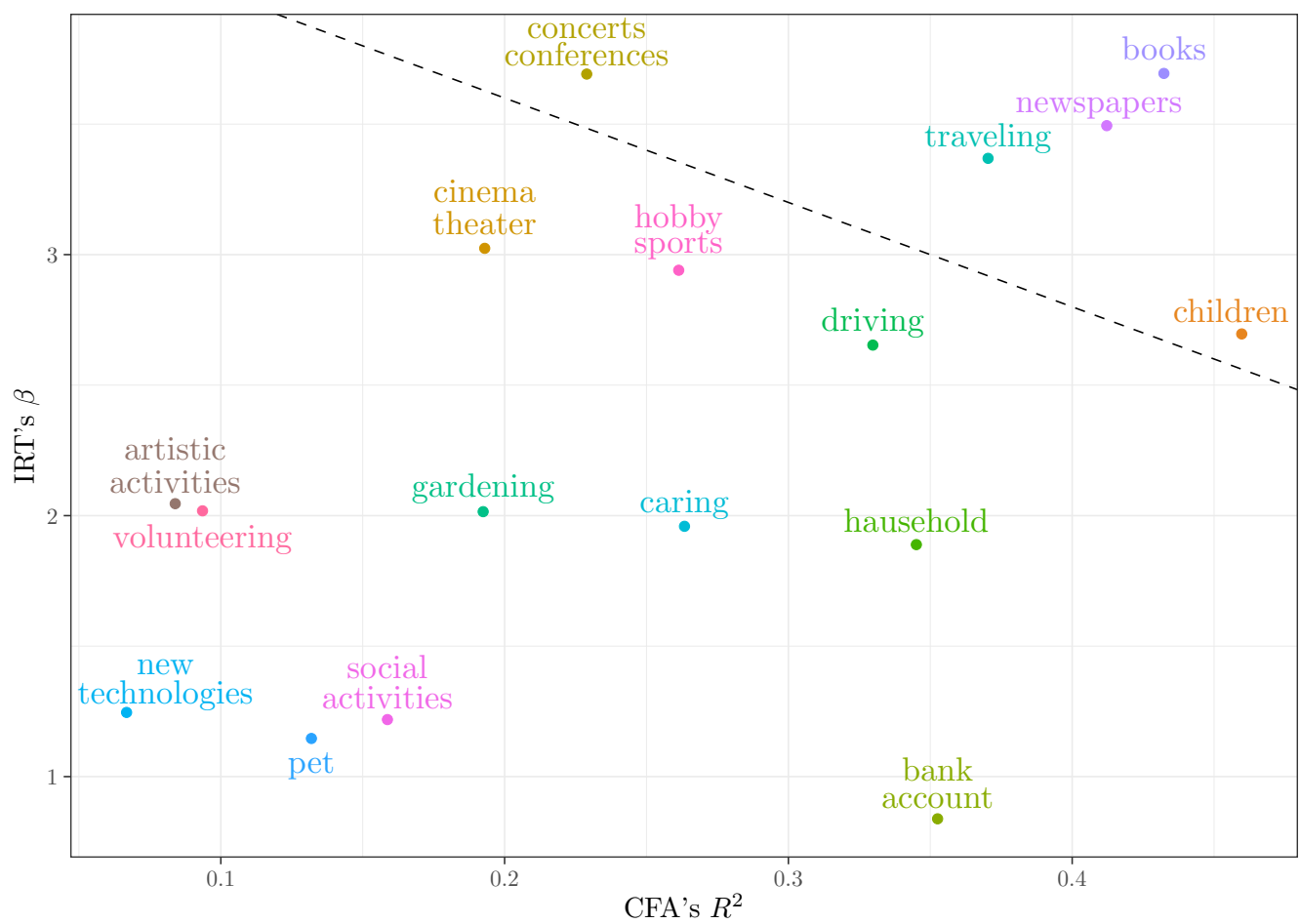

Supplement: Supplementary file 2 — Supplementary file2 (PDF 35 KB) [file 40520_2023_2561_MOESM2_ESM.pdf]
